# Natural Peptides with Potential Applications in Drug Development, Diagnosis, and/or Biotechnology

**DOI:** 10.1155/2012/757838

**Published:** 2012-08-09

**Authors:** Mirian A. F. Hayashi, Frédéric Ducancel, Katsuhiro Konno

**Affiliations:** ^1^Laboratory of Molecular Pharmacology, Departamento de Farmacología, Universidade Federal de São Paulo (Medical School of São Paulo), São Paulo, SP, Brazil; ^2^Responsable du Laboratoire d'Ingénierie des Anticorps pour la Santé Head of the Laboratory of Antibody Engineering for Health (CEA/iBiTecS/SPI/LIAS), CEA de Saclay, Bt 152, 91191 Gif-sur-Yvette Cedex, France; ^3^Institute of Natural Medicine, University of Toyama, 2630 Sugitani, Toyama-shi, Toyama 930-0194, Japan

Natural peptides are central and crucial in many physiological processes playing either direct or indirect roles. Peptides are short linear chains of up to fifty amino acid residues, stabilized or not by disulphide bonds. They occur naturally in all living beings and exert highly specific biological activities, whose specificity is mainly based on and dependent on their primary sequence and, ultimately, to their conformational structure. The primary function of most peptides is the cell signalling role aiming to translate and deliver the biochemical “message” that triggers structural, molecular, cellular, and eventually biological effects. Thus, peptides can play roles as agonists, antagonists, modulators, mediators, hormones, effectors, cofactors, activators, stimulators, and so on.

Also, many peptides can act directly as enzyme inhibitors or as antimicrobial compounds with possible activity on biological membranes, although with no necessary membrane lipid bilayer permeabilisation ability, acting by interfering with metabolism and targeting cytoplasmic components. They are also potentially antigenic compounds and several other peptides are used as pathological biomarkers, since they can be easily and specifically detected and quantified in various biological fluids. 

Based on the huge variety of mode of actions and physiological/pathological roles played by the peptides, in general, their structural and functional relationship has been widely studied by scientific researchers. Their functional roles, their reduced size, their low immunogenicity, their stability, in addition to the recent development of powerful strategies for chemical synthesis and/or recombinant expression, have given to the peptides the status of the most promising family of compounds with potential application for human diagnosis and therapy. Furthermore, their scaffold can been engineered to design compounds with modified biochemical, functional, or biophysical properties, allowing their labelling for *in vivo* imaging and vectorization applications, or also to functionalize nanoparticles.

This special issue aims to gather a recent set of six original articles that mainly further emphasizes the molecular diversity and the variety of mode of action of natural peptides.

Thus, C. Kairane and colleagues, from Estonia (Faculty of Medicine of University of Tartu), have examined the influence of the replacement of *γ*-Glu moiety to *α*-Glu in two gluthatione- (GSH-) related tetrapeptides UPF1 (Tyr (Me)-*γ*-Glu-Cys-Gly) and UPF17 (Tyr (Me)-*α*-Glu-Cys-Gly) in the antioxidative defense system in a human erythroleukemia K562 cell line. By monitoring the effects in these K562 cells via measurements of the cytosolic superoxide dismutase CuZnSOD activity and variations of intracellular GSH levels, followed by addressing the question of the stability of these two peptides against the action of the *γ*-glutamyltranspeptidase (GGT), allowed to the authors to open promising perspectives for the usage of GSH analogues as regulators of the oxidative status of cells. In fact, UPF1 was shown to be resistant to the degradation by GGT. Nonetheless, attention was brought to the fact that UPF1/GSH and UPF17/*α*-GSH have paradoxal effects, suggesting that the effective antioxidative character of peptides is not depend solely on the reactivity of the thiol group, but it might also be dependent of other functional groups and on the spatial structure of peptides.

The short communication by L. B. Bondarenko and V. M. Kovalenko aimed at investigating the potential effect of pyrazinamide on the type II collagen amino acid composition. Indeed, pyrazinamide is a drug classically used for tuberculosis treatment, and the establishment of its effect on a so important cell structural protein is clearly of worth. A dose-dependent quantitative and qualitative effect of pyrazinamide on the male rat extracellular matrix cartilage type II collagen amino acid composition was demonstrated, but additional studies are now necessary to precise and complete this preliminary study.

D. J. Sánchez-González and colleagues, from Mexico, provided to the readers of this special issue a review article on platelet-rich plasma peptides, revealing the central and important roles of these nonnuclear cellular fragments in mammals. Indeed, platelets are characterized by an important role on proteins and peptides synthesis, whose pattern and release in the plasma seems to be modulated in response to different cellular activations. Numerous peptidic growth factors present in the platelet-rich plasma are listed and their activities are also described. Also, the content in bioactive molecules, among which several peptides, present in the alpha granules of platelets are described, and their classification accordingly to their general known activity is shown. Finally, the therapeutic potential of several plasma-derived plasma peptides and their actual clinical status are presented, shedding some light on their potential use in both tissue repair and regenerative medicine.

The K. Wong group's article, from United Kingdom, consisted in a meta-analysis of the existing literature about the therapeutic effects of glucagon-like peptide-1 (GLP-1) agonist in the treatment of heart failure due to ischaemia. The leading cause of systolic heart failure is myocardial ischemia, resulting in the lack of chemical energy transfer from the metabolism of carbon fuels to the contractile work. Thus, metabolic modulators are able to improve the cardiac energetics by altering the substrate from free fatty acids to glucose. This shift results in an optimization of the metabolic efficiency of the heart. The GLP-1 agonist is among these metabolic modulator agents. This comprehensive review of medical literature (including information on preclinical or clinical trials) gives an overall estimate of the therapeutic effectiveness of using GLP-1 agonist in heart failure.

And, in a different topic, the review article presented by M. S. Akhtar and colleagues, from Pakistan, illustrates the particular interest that represents antimicrobial peptides as infection imaging agents. Indeed, differentiation between infection and inflammation by nuclear techniques using radiolabeled compounds is usually difficult. In this review, the authors describe and discuss the merits and demerits that can be attributed to specific radiotracers such as antimicrobial peptides compared to radiolabeled antibiotics for infection localization. Thus, antimicrobial peptides seems to be more specific agents for localizing infections, as they bind specifically to bacterial cell membranes. In fact, gram positive and negative bacteria, *Candida albicans* and also *Aspergillus fumigatus* infections are detected by such tracers. Furthermore, the use of these radiolabeled peptides for monitoring the efficacy and duration of antibiotic treatments is also proposed.

Venom fluids from venomous animals are complex mixtures of several hundreds of components, among which including a number of peptides reticulated or not by disulfide bridges, or sometimes posttranslationaly modified by for instance amidation or phosphorylation. Classically, they bind with high affinity and specificity to different target proteins such as enzymes, ion channels, and receptors. Consequently, they constitute useful and powerful tools for physiological, biochemical and pharmacological studies, supporting further progress in the understanding of the sophisticated relationships between the main biological molecular actors. Interestingly, several of these natural peptides are either the therapeutic molecules by themselves or they have inspired the design of synthetic chemical small molecule drugs. Thus the importance of performing an inventory of the existing natural molecular biodiversity is unquestionable. Classically, mass spectrometry analysis and/or precursors cloning followed by sequencing are the most frequently employed techniques. Also, several groups have successfully applied the next generation sequencing (NGS) strategies, initially used in genome elucidation, to perform exhaustive transcriptomic studies. Actually, T. Kubo's group, from Japan, described the identification of a large variety of venom bioactive peptides by sequencing of cDNA library clones isolated from the Chilean common tarantula *Grammostola rosea* venom gland. The cDNA sequences analysis of about 1,500 clones out of 4,000 clones allowed the identification of 48 novel toxin-like peptides (GTx1 to GTx7, and GTx-TCTP and GTx-CRISP), and among them 24 toxins are ICK motif peptides, 11 peptides are MIT1-like peptides, and 7 are ESTX-like peptides. Peptides similar to JZTX-64, aptotoxin, CRISP or TCTP were also described. Moreover, GTx-CRISP is the first CRISP-like protein identified from the arthropod venom, demonstrating once more the power of applying ESTs techniques to cDNA library to the discovery of novel peptide sequences with potential application in biomedicine.

Together, these articles composing this special issue papers provide to the readers a new and recent set of information on bioactive peptide studies, either in form of original papers or as concise review articles. The common motivation of these different publications is to illustrate the high therapeutic or diagnostic potential associated to the use of natural peptides, or to the design of new drugs inspired in the natural biodiversity of sequences and their wide biological roles.

*Mirian A. F. Hayashi* *Mirian A. F. Hayashi* *Frédéric Ducancel* *Frédéric Ducancel* *Katsuhiro Konno* *Katsuhiro Konno*

